# Allele-specific marker development and selection efficiencies for both flavonoid 3′-hydroxylase and flavonoid 3′,5′-hydroxylase genes in soybean subgenus *soja*

**DOI:** 10.1007/s00122-013-2063-3

**Published:** 2013-03-06

**Authors:** Yong Guo, Li-Juan Qiu

**Affiliations:** The National Key Facility for Crop Gene Resources and Genetic Improvement (NFCRI), Institute of Crop Science, Chinese Academy of Agricultural Sciences, No.12 Zhongguancun South Street, Haidian District, 100081 Beijing, People’s Republic of China

## Abstract

Color is one of the phenotypic markers mostly used to study soybean (*Glycine max* L. Merr.) genetic, molecular and biochemical processes. Two P450-dependent mono-oxygenases, flavonoid 3′-hydroxylase (F3′H; EC1.14.3.21) and flavonoid 3′,5′-hydroxylase (F3′5′H, EC1.14.13.88), both catalyzing the hydroxylation of the B-ring in flavonoids, play an important role in coloration. Previous studies showed that the *T* locus was a gene encoding F3′H and the *W1* locus co-segregated with a gene encoding F3′5′H in soybean. These two genetic loci have identified to control seed coat, flower and pubescence colors. However, the allelic distributions of both *F3′H* and *F3′5′H* genes in soybean were unknown. In this study, three novel alleles were identified (two of four alleles for *GmF3′H* and one of three alleles for *GmF3′5′H*). A set of gene-tagged markers was developed and verified based on the sequence diversity of all seven alleles. Furthermore, the markers were used to analyze soybean accessions including 170 cultivated soybeans (*G. max*) from a mini core collection and 102 wild soybeans (*G. soja*). For both *F3′H* and *F3′5′H*, the marker selection efficiencies for pubescence color and flower color were determined. The results showed that one *GmF3′H* allele explained 92.2 % of the variation in tawny and two *gmf3′h* alleles explained 63.8 % of the variation in gray pubescence colors. In addition, two *GmF3′5′H* alleles and one *gmF3′5′h* allele explained 94.0 % of the variation in purple and 75.3 % in white flowers, respectively. By the combination of the two loci, seed coat color was determined. In total, 90.9 % of accessions possessing both the *gmf3′h*-*b* and *gmf3′5′h* alleles had yellow seed coats. Therefore, seed coat colors are controlled by more than two loci.

**Electronic supplementary material:**

The online version of this article (doi:10.1007/s00122-013-2063-3) contains supplementary material, which is available to authorized users.

## Introduction

Flower, seed and fruit colors are important in the ecology of plants and in their ability to attract pollinators and seed-dispersing organisms (Grotewold 2006; Mol et al. 1998). In addition, color is one of the most widely used phenotypic markers in the study of genetic, molecular and biochemical processes, due to their easy recognizability (Holton and Cornish 1995; Koes et al. 2005). The pigments that color most flowers, fruits and seeds are of three types: betalains, carotenoids, and flavonoids (Tanaka et al. 2008). Of these, the flavonoids have been studied most in the context of pigmentation, and are well conserved among higher plants (Hichri et al. 2011; Lepiniec et al. 2006; Winkel-Shirley 2001). Flavonoids mainly consist of anthocyanins, chalcone, flavone, flavonol, flavanone and isoflavonoids. Of these, anthocyanins are broadly distributed in flowering plants and predominantly contribute to flower, fruit and seed colors (Han et al. 2010). Beside their multiple roles in coloring plant organs, flavonoids are of great interest for plant adaptation to the environment and for human health (Halbwirth 2010; Harvaux and Kloppstech 2001; Li et al. 1993; Pourcel et al. 2007; Scalbert et al. 2005; Winkel-Shirley 2002).

The biosynthetic pathway of flavonoids is well established and many of the structural and some of the regulatory genes have been cloned in several model plants including *Arabidopsis*, maize (*Zea mays*), snapdragon (*Antirrhinum majus*) and *Petunia* (Hichri et al. 2011; Holton and Cornish 1995; Mol et al. 1998; Winkel-Shirley 2001). The precursors for the synthesis of all flavonoids are malonyl-CoA and *p*-coumaroyl-CoA. Chalcone synthase catalyzes the stepwise condensation of three acetate units from malonyl-CoA with p-coumaroyl-CoA to yield tetrahydroxychalcone. The synthesis of colored anthocyanins from tetrahydroxychalcone is catalyzed by a series of enzymes, including chalcone isomerase, flavanone 3-hydroxylase, flavonoid 3′-hydroxylase (F3′H), flavonoid 3′,5′-hydroxylase (F3′5′H), dihydroflavonol 4-reductase, anthyocyanidin synthase and uridine diphosphate glucose: flavonoid-3-O-glucosyltransferase (Holton and Cornish 1995). Among these, F3′H and F3′5′H, which are microsomal cytochrome P450-dependent mono-oxygenases that require NADPH as a co-factor, are key enzymes to hydroxylate the B-ring in flavonoids (Forkmann 1991). As the hydroxylation pattern of the B-ring in flavonoids plays an important role in coloration and determines their stability and antioxidant capacity, the activity of F3′H and F3′5′H strongly influences flower and seed coloration (de Vetten et al. 1999; Han et al. 2010; Holton et al. 1993; Ishiguro et al. 2011; Moreau et al. 2012; Schoenbohm et al. 2000; Zabala and Vodkin 2003). To date, the *F3′H* and *F3′5′H* genes have been isolated from many plant species, including *Petunia* (Brugliera et al. 1999; Holton et al. 1993), *Arabidopsis* (Schoenbohm et al. 2000), soybean (Toda et al. 2002; Zabala and Vodkin 2007), grape (Bogs et al. 2006; Falginella et al. 2010), pea (Moreau et al. 2012), maize (Sharma et al. 2011), and apple (Han et al. 2010). Furthermore, manipulation of *F3′H* and *F3′5′H* has been effective in the genetic engineering of floral crops to develop new genotypes with novel flower colors for ornamental purposes (Nakatsuka et al. 2007; Tanaka et al. 2010).

The chemistry and genetics of pigmentation in seeds and flowers has also been extensively studied in soybean (Palmer and Stelly 1979; Stephens and Nickell 1992; Todd and Vodkin 1993), Thus far, alleles of at least five loci (*I, T, W1, R,* and *O*) are known to function epistatically to control seed coat pigmentation, six loci (*W1, W2, W3, W4, Wm,* and *Wp*) control flower pigmentation and two loci (*T* and *Td*) control pubescence color in soybean (Palmer et al. 2004; Stephens and Nickell 1992). So far, the molecular biology of these soybean loci has been studied but only a few loci have been identified at the molecular level due to the complexity of the soybean genome. Classic genetics showed that *T* conferred brown and *t* conferred gray pubescence color. Further, *T* generally darkens hilum and/or seed coat color in combination with genotypes at the *I*, *W1*, *R* and *O* loci (Palmer et al. 2004). Cloning and mapping of the soybean *sf3′h1* genomic and cDNA sequences identified the *F3′H* gene at the *T* locus on linkage group C2 (chromosome 6) (Toda et al. 2002; Zabala and Vodkin 2003). Silencing of *sf3′h1* resulted in decreased levels of quercetin and loss of the tawny pigmentation in pubescence (Nagamatsu et al. 2009; Nagamatsu et al. 2007), suggesting that the *sf3′h1* gene is responsible for the production of quercetin and involved in the control of pigmentation in pubescence. Moreover, the relationship between the *T* locus and chill-tolerance has also been studied (Takahashi and Asanuma 1996; Takahashi et al. 2005; Toda et al. 2012), indicating the importance of the soybean *F3′H* gene in pigment biosynthesis and physiological function. The *W1* locus has a pleiotropic effect on flower and hypocotyl color, in which soybean accessions with purple/white flowers have purple/green hypocotyls. It was confirmed that *W1* locus co-segregated with a gene encoding F3′5′H by using near isogenic lines (Zabala and Vodkin 2007). In addition, the light-purple flower of the wild soybean B09121 is controlled by a new allele of the *W1* locus encoding F3′5′H (Takahashi et al. 2010), suggesting that more alleles of these genes remain to be identified.

The sequences of *F3′H* and *F3′5′H* have been cloned from some soybean accessions; however, no functional markers and allelic diversity analysis of these genes have been published so far. Due to the abundant variation in the soybean genome, whether *F3′H* and *F3′5′H* have other alleles and their distribution in accessions need to be revealed. The core collection and mini core collection of soybean provide an effective platform for genetic diversity studies, novel gene identification, and allele distribution analysis (Song et al. 2010; Wang et al. 2006). In this study, three novel alleles, two of four alleles for *GmF3′H* and one of three alleles for *GmF3′5′H*, were found on the basis of the sequence variation of these two genes in different soybean accessions. A set of gene-tagged markers were developed and verified. The genetic effects of *GmF3′H* and *GmF3′5′H* were also detected by genotyping 272 accessions including part of the mini core collection of cultivated soybeans and annual wild soybeans.

## Materials and methods

### Plant materials

The collection of annual wild soybeans (*Glycine soja*) and a partial mini core collection of cultivated soybeans (*Glycine max*) including elite cultivars and local landraces described previously (Li et al. 2008) were obtained from the National Genebank at the Institute of Crop Science, Chinese Academy of Agricultural Sciences. Two kinds of pubescence colors and flower colors, and five kinds of seed coat colors (Supplemental Fig. 1) were classified according to Qiu and Chang (2006).

### RNA extraction, cDNA cloning and sequencing

Total RNA from frozen leaves of soybean ecotype Willimas82 and Zhonghuang13 was extracted using TRIzol reagent (Invitrogen, USA). To eliminate the contamination of genomic DNA, total RNA was treated with RNase-free DNase (TaKaRa, Japan). The cDNA was synthesized using the ReverTra Ace qPCR RT kit (Toyobo, Japan) in a reaction of 20 μL. The full-coding cDNA clones of *GmF3′H* and *GmF3′5′H* were generated by RT-PCR and the primers used to amplify the coding sequences were listed in Supplemental Table 1. The coding sequences were determined after cloning into the pBluescript SK + (pBS) vector, and confirmed by comparison with the corresponding genomic sequences.

### Multiple sequence alignments and phylogenetic tree building

To generate the phylogenetic tree of F3′Hs and F3′5′Hs from different organisms, the amino-acid sequences of *F3′Hs* and *F3′5′Hs* identified previously were collected from the NCBI database (http://www.ncbi.nlm.nih.gov). Additional amino-acid sequences of F3′Hs were from: *Arabidopsis thaliana* (AAG16746), *Petunia* × *hybrida* (AAD56282), *Vitis vinifera* (CAI54278), *Brassica napus* (ABC58723), *Antirrhinum majus* (ABB53383), *Malus* × *domestica* (ACR14867), *Zea mays* (AEF33624), *Sorghum bicolor* (AAV74195), and *Oryza sativa* (AAP52914). Additional amino-acid sequences of F3′5′Hs were: *Petunia* × *hybrida* (CAA80265), *Gentiana scabra* (BAE86871), *Pisum sativum* (ADW66160), *Antirrhinum kelloggii* (BAJ16328), *Vitis vinifera* (CAI54277), *Solanum tuberosum* (AAV85470), *Solanum lycopersicum* (ADC80513), *Phalaenopsis hybrid* (AAZ79451), *Dendrobium moniliforme* (AEB96145), and *Hordeum vulgare* (BAK02913). All sequences were aligned with ClustalX and improved manually. Phylogenetic trees were constructed by the neighbor-joining method using MEGA 4.0 and internal branch support was estimated with 1,000 bootstrap replicates.

### Genomic DNA isolation and PCR for sequencing

Genomic DNA was isolated from soybean leaves using the DNeasy Plant Mini Kit (Qiagen, USA) and used at 10–20 ng per PCR amplification. PCR was carried out using KOD-Plus-Neo (Toyobo, Japan) according to the manufacturer’s recommendations in a PTC-200 Thermocycler (MJ Research/Bio-Rad, USA). The PCR reaction cycles were as follows: 1 cycle (94 °C, 3 min), 36 cycles (94 °C, 15 s; 60 °C, 15 s; 68 °C, 30 s), and a final extension step (68 °C, 8 min). PCR products were analyzed by gel electrophoresis to verify the size and ensure specific amplification, and then sequenced after isolation. The primers used for amplification and sequencing are listed in Supplementary Table 1.

### Marker development

Gene-tagged markers were developed based on the sequence variation of different alleles. CAPs markers F3′H-ApoI and F3′5′H-HphI were developed based on single-nucleotide deletion of adenine at position 973 of the *GmF3′H* coding sequence and single-nucleotide substitution at position 1424 of the *GmF3′5′H* coding sequence. The dCAP markers F3′H-MjaIV and F3′H-EcoNI were developed based on the artificial introduction of a restriction enzyme-recognition site at the end of the forward primer. The InDel marker F3′5′H-In was developed based on the 53-bp difference between the two alleles of *GmF3′5′H*.

### Genotype analysis

PCR was carried out in the PTC-200 Thermocycler using the genomic DNA of all materials. PCR products were directly separated on 2 % agarose gels for the InDel marker or digested with the appropriate restriction enzymes for the CAPS and dCAPS markers. The samples were incubated at the temperature recommended by the manufacturer for >1 h, and then separated on 2 % agarose gels stained with ethidium bromide followed by photography.

## Results

### Cloning of the *GmF3′H* and *GmF3′5′H* genes from soybean and phylogenetic analysis

The full-length protein-coding sequences for *GmF3′H* (*Glyma06g21920*) from soybean ecotype Williams 82 and *GmF3′5′H* (*Glyma13g04210*) from Zhonghuang13 were obtained by RT-PCR. Sequence comparison of the cloned coding sequences and the published genomic sequence indicated the presence of three exons and two introns in these genes. Sequence analysis revealed a putative polypeptide consisting of 513 amino-acids from the coding sequence of *GmF3′H*, and one of 509 amino-acids from *GmF3′5′H*.

**Fig. 1:**
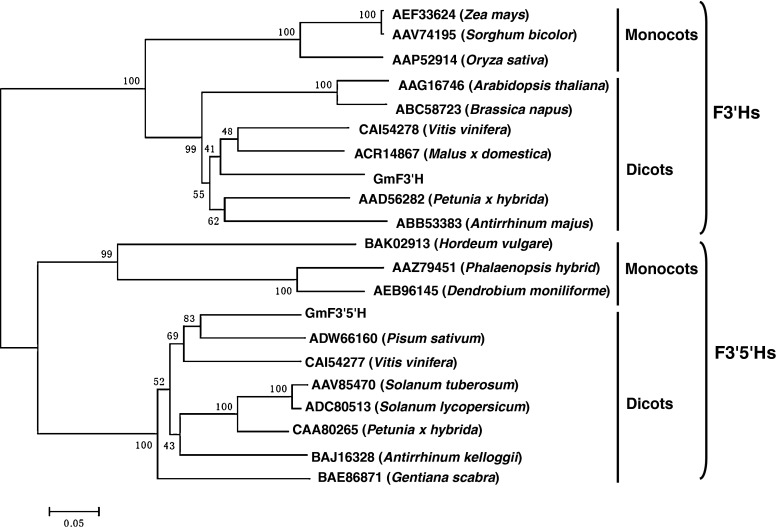
Phylogenetic tree of F3′Hs and F3′5′Hs proteins from soybean and other species. Neighbor-joining tree of F3′Hs and F3′5′H proteins from soybean and other species generated using MEGA 4.0. The numbers on each node are bootstrap values, which indicate the percentage of bootstrap replicates that support this node out of 1,000 samples. Branch lengths are proportional to the estimated number of amino-acid substitutions. *Scale bar* indicates the estimated amino-acid substitutions per site. F3′H superfamily; *Arabidopsis thaliana* (AAG16746), *Petunia x hybrida* (AAD56282), *Vitis vinifera* (CAI54278), *Brassica napus* (ABC58723), *Antirrhinum majus* (ABB53383), *Malus* × *domestica* (ACR14867), *Zea mays* (AEF33624), *Sorghum bicolor* (AAV74195), and *Oryza sativa* (AAP52914). F3′5′H superfamily; *Petunia* × *hybrida* (CAA80265), *Gentiana scabra* (BAE86871), *Pisum sativum* (ADW66160), *Antirrhinum kelloggii* (BAJ16328), *Vitis vinifera* (CAI54277), *Solanum tuberosum* (AAV85470), *Solanum lycopersicum* (ADC80513), *Phalaenopsis hybrid* (AAZ79451), *Dendrobium moniliforme* (AEB96145), *Hordeum vulgare* (BAK02913)

To study the relationship of GmF3′H and GmF3′5′H with other F3′H/F3′5′H proteins, phylogenetic analysis was carried out using the deduced amino-acid sequences of GmF3′H and GmF3′5′H with other known plant-specific flavonoid hydroxylase proteins from *Arabidopsis*, *Petunia*, grape, rapeseed (*Brassica napus*), snapdragon, apple, maize, grain sorghum (*Sorghum bicolor*), rice (*Oryza sativa*), and so on. The phylogenetic tree was separated into two large groups, with all F3′Hs and F3′5′Hs clustered in the different clades (Fig. 1). GmF3′H/GmF3′5′H resided in the same clade as other dicots, and were separated from the monocots such as maize, grain sorghum, rice, *P. hybrid* and *D. moniliforme* (Fig. 1). Moreover, F3′H/F3′5′H from the most closely related species formed closely related clades, such as *Arabidopsis* and rapeseed, maize and grain sorghum.

### Allelic diversity in the *GmF3′H* and *GmF3′5′H* genes

**Fig. 2:**
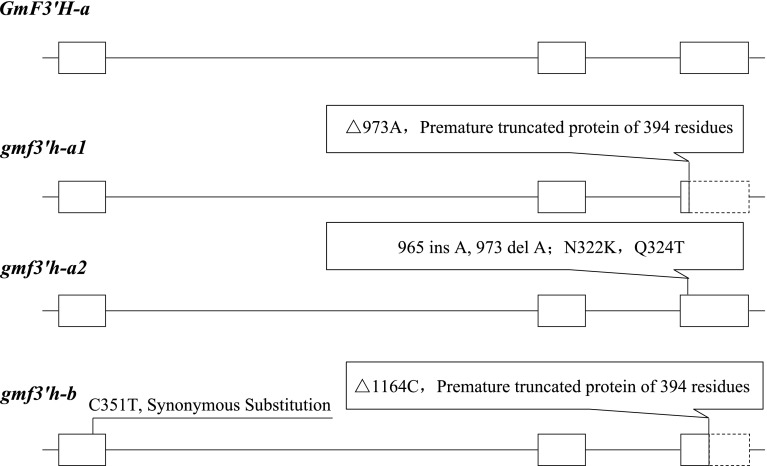
*GmF3′H* gene structure and polymorphisms in different alleles. The *gmf3′h*-*a1* allele presents a single base deletion of adenine at coding sequence position 973 relative to the start codon. The deletion creates a premature stop codon and a truncated protein of 394 residues compared to the reference GmF3′H protein of 513 residues. The *gmf3′h*-*a2* allele presents a single base insertion of an adenine at position 965 as well as a single base deletion of adenine at position 973, resulting in N322 K and Q324T changes. The *gmf3′h*-*b* allele presents a single base deletion of a cytosine at position 1164. This cytosine deletion resulted in a frame-shift that prematurely truncated the protein of 394 residues. *Boxes* represent exons; *lines between boxes* represent introns; *boxes with dashed lines* represent exons with untranslated regions in predicted proteins from different alleles

**Fig. 3:**
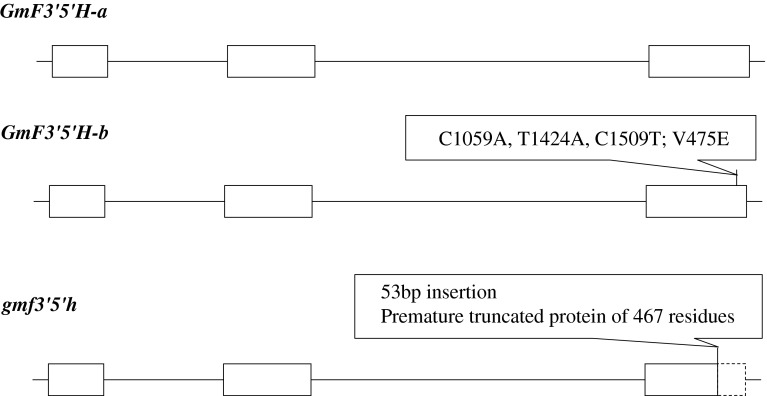
*GmF3′5′H* gene structure and polymorphisms in different alleles. The *GmF3′5′H*-*b* allele had three nucleotide substitutions in exon 3 (C1059A, T1424A, C1509T): two synonymous and one non-synonymous substitution (V475E). The *gmf3′5′h* allele had a 53-bp fragment insertion at coding sequence position 1352 relative to the start codon, resulting in a prematurely truncated amino-acid sequence of 467 residues compared to the reference GmF3′5′H protein of 501 residues. *Boxes* represent exons; *lines between boxes* represent introns; *boxes with dashed lines* represent exons with untranslated regions in predicted proteins from different alleles

To study the sequence diversity of the coding regions in the *GmF3′H* *and GmF3′5′H* genes, 30 soybean accessions (12 modern elite cultivars, ten local landraces and eight annual wild soybeans) were used for sequence analysis, and four alleles of *GmF3′H* and three alleles of *GmF3′5′H* were found (Table 1; Figs. 2, 3).

**Table 1:** Names, resources, origin, phenotypes and allele types of 30 soybean cultivars selected for sequencing

No.	Cultivar/Line	Germplasm resource	Origin	Pubescence color	Flower color	*GmF3′H* ^a^	*GmF3′5′H* ^b^
1	Williams 82	Cultivars	USA	Tawny	White	1	3
2	Wenfeng7	Cultivars	Shandong, China	Gray	White	4	3
3	Union	Cultivars	USA	Tawny	White	1	3
4	Zhongpin03-5373	Cultivars	Beijing, China	Gray	Purple	2	1
5	Zhonghuang13	Cultivars	Beijing, China	Gray	Purple	2	1
6	Jidou12	Cultivars	Hebei, China	Gray	Purple	2	2
7	Ji NF58	Cultivars	Hebei, China	Tawny	White	1	3
8	Suinong14	Cultivars	Heilongjiang, China	Gray	Purple	4	1
9	Suinong20	Cultivars	Heilongjiang, China	Gray	White	4	3
10	Zheng92116	Cultivars	Henan, China	Gray	Purple	4	1
11	Shang951099	Cultivars	Henan, China	Tawny	Purple	3	2
12	Baiqiu1	Cultivars	Fujian, China	Tawny	White	1	3
13	Peking	Landraces	USA	Tawny	White	1	3
14	Dahedou	Landraces	Hebei, China	Tawny	White	1	2
15	Hanyuanbalixiaoheidou	Landraces	Sichuan, China	Tawny	Purple	1	2
16	Yingdehedou	Landraces	Guangdong, China	Tawny	Purple	3	2
17	Changshanidou	Landraces	Hunan, China	Tawny	Purple	1	2
18	Huipizhiheidou	Landraces	Shanxi, China	Tawny	Purple	1	2
19	Zhechengxiaohongdou	Landraces	Henan, China	Tawny	Purple	1	2
20	Pixiansilicao	Landraces	Jiangsu, China	Gray	Purple	4	1
21	Xiataizimoshidou	Landraces	Hebei, China	Gray	Purple	1	2
22	Heidou	Landraces	Shanxi, China	Tawny	Purple	1	1
23	ZYD03687	Wild soybean	Henan, China	Gray	Purple	1	1
24	ZYD00401	Wild soybean	Heilongjiang, China	Tawny	Purple	1	1
25	ZYD04186	Wild soybean	Jiangsu, China	Tawny	Purple	1	1
26	ZYD02878	Wild soybean	Shanxi, China	Tawny	Purple	1	1
27	ZYD04734	Wild soybean	Guizhou, China	Tawny	Purple	1	1
28	ZYD04569	Wild soybean	Zhejiang, China	Tawny	Purple	1	1
29	ZYD04638	Wild soybean	Jiangxi, China	Tawny	Purple	1	1
30	ZYD02738	Wild soybean	Hebei, China	Tawny	Purple	1	1

^**a**^For *GmF3′H*, 1, 2, 3 and 4 represent the *GmF3′H*, *gmf3′h*-*a1*, *gmf3′h*-*a2* and *gmf3′h*-*b* alleles

^**b**^For *GmF3′5′H*, 1, 2 and 3 represent the *GmF3′5′H*-*a*, *GmF3′5′H*-*b*, and *gmf3‘5′h* alleles

In the *gmf3′h*-*a1* allele from Zhongpin03-5373, Zhonghuang13 and Jidou-12, a single base deletion of an adenine at coding sequence position 973 relative to the start codon resulted in a premature stop codon and a truncated protein of 394 residues (Fig. 2). In the *gmf3′h*-*a2* allele from Shang951099 and Yingdehedou, a single base insertion of an adenine at coding sequence position 965 as well as a single base deletion of an adenine at position 973 only resulted in N322K and Q324T changes for no frame-shift with the single base deletion right after the single base insertion. In the *gmf3′h*-*b* allele from Wenfeng7, Suinong14, Suinong20, Zheng92116 and Pixiansilicao, a single base deletion of a cytosine was identified at position 1164. This deletion resulted in a frame-shift that prematurely truncated the protein after only six amino-acids. The *GmF3′H* alleles in 20 other accessions (designated *GmF3′H*) were the same as the reference Williams 82 sequence and encoded the full-length amino-acids.

In the *gmf3′5′h* allele in Williams 82, Wenfeng7, Union, JiNF58, Suinong20, Baiqiu1 and Peking, a 53-bp fragment was inserted at coding sequence position 1352 relative to the start codon, resulting in a prematurely truncated amino-acid sequence of 467 residues (Fig. 3). In the other cultivars/lines, the *GmF3′5′H* alleles from 14 accessions (designated *GmF3′5′H*-*a*) were the same as the sequence of Zhonghuang13 and encoded the full-length amino-acids while those in another nine accessions (*GmF3′5′H*-*b*) had three nucleotide substitutions in exon 3 (C1059A, T1424A, C1509T) resulting in two synonymous and one non-synonymous substitution (V475E).

### Development of gene-tagged markers

**Fig. 4:**
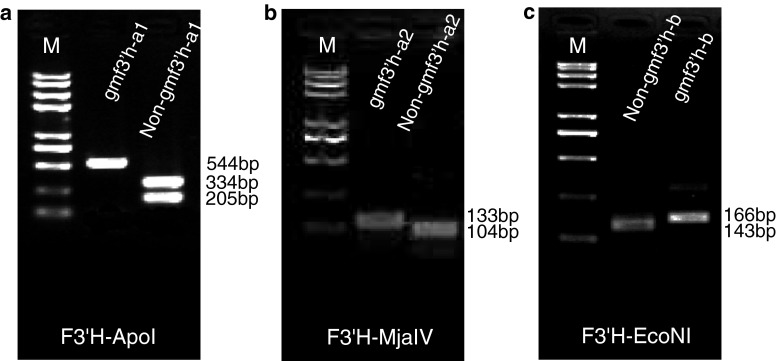
Polymorphisms revealed by three gene-tagged markers of *GmF3′H*. The F3′H-ApoI marker distinguished *gmf3′h*-*a1* and non-*gmf3′h*-*a1* alleles (**a**); the F3′H-MjaIV marker distinguished *gmf3′h*-*a2* and *non*-*gmf3′h*-*a2* alleles (**b**); the F3′H-EcoNI marker distinguished *gmf3′h*-*b* and *non*-*gmf3′h*-*b* alleles (**c**)

Molecular markers were designed to distinguish the alleles of *GmF3′H* and *GmF3′5′H* (Table 2). For the *gmf3′h*-*a1* and *gmf3′h*-*a2* alleles, a CAPS marker designated as F3′H-ApoI was generated based on the single nucleotide deletion of adenine at coding sequence position 973. At the deletion site, the *GmF3′H* and *gmf3′h*-*b* alleles could be recognized by *Apo*I, whereas the *gmf3′h*-*a1* and *gmf3′h*-*a2* alleles could not be recognized by this enzyme. The PCR results showed that the products were 540 bp in length as expected, and the PCR products of the *GmF3′H* and *gmf3′h*-*b* alleles were digested into two fragments (322 and 208 bp) by *Apo*I, whereas the products of the *gmf3′h*-*a1* and *gmf3′h*-*a2* alleles were not cleaved. Hence, the F3′H-ApoI marker could be used to identify both the *gmf3′h*-*a1* and *gmf3′h*-*a2* alleles (Fig. 4a). To distinguish the *gmf3′h*-*a1* and *gmf3′h*-*a2* alleles, a dCAPs marker was developed, since no restriction enzyme recognition sites were detected at the insertion site of adenine at position 965. A restriction enzyme recognition site (GTNNAC, *Mja*IV) was artificially introduced at the end of the forward primer, containing two mismatched nucleotides (GT; Table 2). The PCR results showed that the expected DNA fragments (133/132 bp) were easily amplified using materials with *gmf3′h*-*a1* and *gmf3′h*-*a2* alleles. The PCR products were then digested by the *Mja*IV enzyme. The product of the *gmf3′h*-*a1* allele was digested, and a 105-bp fragment was obtained. On the other hand, the PCR product of the *gmf3′h*-*a2* allele could not be cleaved by *Mja*IV (Fig. 4b). We concluded that the F3′H-MjaIV marker can be used to separate the two alleles. For the *gmf3′h*-*b* allele, a dCAPS marker was also developed due to the absence of restriction enzyme recognition sites at the deletion site. Similarly, the recognition site of *Eco*NI (CCTNNNNNAGG) was artificially introduced at the end of the forward primer, containing only one mismatched nucleotide (C; Table 2). The PCR products were amplified and then digested by the *Eco*NI enzyme. The product of the *gmf3′h*-*b* allele could not be digested and gave a 167-bp fragment, while that of the other alleles was digested and a 140-bp fragment was obtained (Fig. 4c).

**Table 2:** Gene-tagged markers for *GmF3′H* and *GmF3′5′H*

Marker Name	Primer (5′–3′)		Product size (bp)	Marker type
F3′H-ApoI	Forward	TCCAACTACAACATCTCACCTTAGAA	205 + 334/538	CAPS/ApoI
	Reverse	CTCAAAGTCATTGCCCCTAACA		
F3′H-MjaIV	Forward	GAATGGGCCATTGCGGAACTAATAGTAAA	27 + 105/133	dCAPS/MjaIV
	Reverse	CAGCTTGTAAGTATGGGAGGTGGGC		
F3′H-EcoNI	Forward	GTGAGATATTTGGCTACCACCTCC	24 + 143/166	dCAPS/EcoNI
	Reverse	CTCAAAGTCATTGCCCCTAACA		
F3′5′H-In	Forward	CCCAACCAATTCTAAGAAATGTAA	342/395	InDel
	Reverse	CCCAACCAATTCTAAGAAATGTAA		
F3′5′H-HphI	Forward	CATAGGAAGAGACCCTGATGTGT	225 + 117/342	CAPS/HphI
	Reverse	CCCAACCAATTCTAAGAAATGTAA

**Fig. 5:**
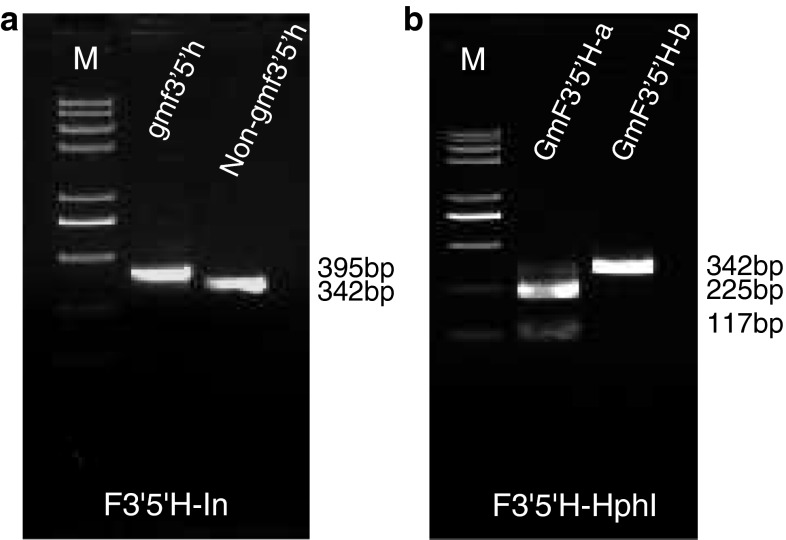
Polymorphisms revealed by two gene-tagged markers of *GmF3′5′H*. The F3′5′H-In marker distinguished *gmf3′5′h* and non-*gmf3′5′h* alleles (**a**); the F3′5′H-HphI marker distinguished *GmF3′5′H*-*a* and *GmF3′5′H*-*b* alleles (**b**)

For the *gmf3′5′h* allele, an InDel marker was developed based on the 53-bp difference between *gmf3′5′h* and *GmF3′5′H*. The PCR results revealed that the GmF3′5′H-In marker successfully differentiated the *gmf3′5′h* and *GmF3′5′H* alleles by the amplification of 395- and 342-bp DNA fragments, respectively, as predicted (Fig. 5a). To distinguish the *GmF3′5′H*-*a* and *GmF3′5′H*-*b* alleles, a CAPS marker, F3′5′H-HphI, was developed based on a single-nucleotide substitution (T to A) at coding sequence position 1424. At the substitution site, the *GmF3′5′H*-*a* allele could be recognized by *Hph*I whereas the *GmF3′5′H*-*b* allele could not. The PCR products were amplified and then digested by *Hph*I. The product of the *GmF3′5′H*-*b* allele could not be digested and gave a 342-bp fragment, while that of the *GmF3′5′H*-*a* allele was digested and 225- and 117-bp fragments were obtained (Fig. 5b).

The developed markers F3′H-ApoI, F3′H-MjaIV, F3′H-EcoNI, F3′5′H-In and F3′5′H-HphI were verified by analysis of allelic diversity using the 30 soybean accessions. The results suggested that the identification of molecular markers was consistent with the sequencing data. Further, these markers can be easily used on a large scale in genotyping basically dependent on the PCR technique.

### Distribution of alleles of *GmF3′H* and *GmF3′5′H* in soybean subgenus *soja*

To determine the genetic constitution of the *GmF3′H* and *GmF3′5′H* alleles in the soybean subgenus *soja*, the developed markers were used to genotype 272 accessions: 170 cultivated soybeans (*G. max*) from mini core collection, consisting of 24 modern elite cultivars and 146 local landraces, and 102 wild soybeans (*G. soja*) (Table 3, Supplemental File). All the wild soybeans contained the *GmF3′H* allele. The ranking of *GmF3′H* allele frequencies in cultivars and landraces was similar, with *GmF3′H* > *gmf3′h*-*b* > *gmf3′h*-*a1* > *gmf3′h*-*a2*. *GmF3′H* was the most common allele in cultivars (45.8 %) and landraces (63.0 %) and *gmf3′h*-*b* (29.2 and 28.1 %, respectively) was the second main allele. With regard to the *GmF3′5′H* locus, the most common allele in wild soybeans was *GmF3′5′H*-*a* (92.2 %). Only six wild soybeans possessed *GmF3′5′H*-*b* and two had *gmf3′5′h*. However, the ranking of *GmF3′5′H* allele frequencies in cultivars and landraces differed from wild soybeans, with *gmf3′5′h* > *GmF3′5′H*-*b* > *GmF3′5′H*-*a*. Nei’s gene diversity index analysis suggested that the diversity of both *GmF3′H* and *GmF3′5′H* was higher in cultivated than in wild soybeans (Table 3).

**Table 3:** Distributions of *GmF3′H* and *GmF3′5′H* alleles and their frequencies among cultivars, landraces, and wild soybeans

Gene locus	Allele type	Partial mini core collection		Wild soybeans
		Elite cultivars	Local landraces	
*GmF3′H*	*GmF3′H*	11 (45.8 %)	92 (63.0 %)	102 (100.0 %)
	*gmf3′h*-*a1*	4 (16.7 %)	9 (6.2 %)	0
	*gmf3′h*-*a2*	2 (8.3 %)	4 (2.7 %)	0
	*gmf3′h*-*b*	7 (29.2 %)	41 (28.1 %)	0
	Nei’s gene diversity index	0.67	0.52	0.00
*GmF3′5′H*	*GmF3′5′H*-*a*	5 (20.8 %)	43 (29.5 %)	94 (92.2 %)
	*GmF3′5′H*-*b*	7 (29.2 %)	50 (34.2 %)	6 (5.9 %)
	*gmf3′5′h*	12 (50.0 %)	53 (36.3 %)	2 (2.0 %)
	Nei’s gene diversity index	0.62	0.66	0.15

### Genetic effects of *GmF3′H* and *GmF3′5′H* genes

Previous studies showed that the *T* locus was a gene encoding F3′H and the *W1* locus co-segregated with a gene encoding F3′5′H in soybean, which belonged to the genetic loci controlling seed coat, flower and pubescence color. The genetic effect of *GmF3′H* alleles on pubescence color and seed coat was estimated (Table 4). The results showed that >80 % of accessions possessing the *GmF3′H* (86.3 %) or *gmf3′h*-*a2* (83.3 %) alleles had a tawny pubescence color while >80 % of those possessing the *gmf3′h*-*a1* (84.6 %) or *gmf3′h*-*b* (83.3 %) alleles had a gray pubescence color. As to seed coat color, ~45.4 % accessions possessing *GmF3′H* had a black seed coat, which was the most widespread seed coat color of the *GmF3′H* allele. However, most accessions possessing *gmf3′h*-*a1, gmf3′h*-*a2* or *gmf3′h*-*b* had a yellow seed coat while few had other seed coat colors. On the other hand, these results also suggested that one *GmF3′H* allele explained 177/192 (92.2 %) of tawny and two *gmf3′h* alleles (*gmf3′h*-*a1* and *gmf3′h*-*b*) explained 51/80 (63.8 %) of gray colors.

**Table 4:** Frequency of allelic variation in *GmF3′H* and pubescence and seed coat color of soybean

Allele type	No. of accessions	Pubescence color		Seed coat color				
		Tawny (%)	Gray (%)	Yellow (%)	Green (%)	Brown (%)	Black (%)	Double (%)
*GmF3′H*	205	177 (86.3)	28 (13.7)	46 (22.4)	16 (7.8)	31 (15.1)	93 (45.4)	19 (9.3)
*gmf3′h*-*a1*	13	2 (15.4)	11 (84.6)	11 (84.6)	0	1 (7.7)	0	1 (7.7)
*gmf3′h*-*a2*	6	5 (83.3)	1 (16.7)	5 (83.3)	0	1 (16.7)	0	0
*gmf3′h*-*b*	48	8 (16.7)	40 (83.3)	38 (79.2)	8 (16.7)	1 (2.1)	0	1 (2.1)
Total	272	192	80	100	24	34	93	21

For the *F3′5′H* locus, 92.3 % of accessions possessing *GmF3′5′H*-*a* and 88.9 % possessing *GmF3′5′H*-*b* had a purple flower while 82.1 % of those possessing *gmf3′5′h* had a white flower (Table 5). With regard to seed coat color, ~54.9 % accessions possessing *GmF3′5′H*-*a* had a black seed coat, which was also the most widespread seed coat color of the *GmF3′5′H* allele. However, the most widespread seed coat color of the *gmf3′5′h* allele was yellow (73.1 %). The relationship between *GmF3′5′H*-*b* and seed coat color was not very close and the frequency of each color was similar. Two *GmF3′5′H* alleles and one *gmF3′5′h* allele explained 187/199 (94.0 %) of purple and 55/73 (75.3 %) of white flowers.

**Table 5:** Frequency of allelic variation in *GmF3′5′H* and flower and seed coat color of soybean

Allele type	No. of accessions	Flower color		Seed coat color				
		Purple (%)	White (%)	Yellow (%)	Green (%)	Brown (%)	Black (%)	Double (%)
*GmF3′5′H*-*a*	142	131 (92.3)	11 (7.7)	32 (22.5)	8 (5.6)	10 (7.0)	78 (54.9)	14 (9.9)
*GmF3′5′H*-*b*	63	56 (88.9)	7 (11.1)	19 (30.2)	9 (14.3)	18 (28.6)	11 (17.5)	6 (9.5)
*gmF3′5′h*	67	12 (17.9)	55 (82.1)	49 (73.1)	7 (10.4)	6 (9.0)	4 (6.0)	1 (1.5)
Total	272	199	73	100	24	34	94	20

When the genetic effect of the *GmF3′H* and *GmF3′5′H* alleles were combined, the results showed that these loci had a high association with seed coat color (Table 6). Sixty-five percent of accessions possessing both *GmF3′H* and *GmF3′5′H*-*a* had a black seed-coat and 90.9 % of those possessing both *gmf3′h*-*b* and *gmf3′5′h* had a yellow seed-coat, contributions higher than the single locus.

**Table 6:** Frequency of allelic variation in *GmF3′H* and *GmF3′5′H* and seed coat color of soybean

Genotype	No. of accessions	Seed coat color				
		Yellow (%)	Green (%)	Brown (%)	Black (%)	Double (%)
*GmF3′H* and *GmF3′5′H*-*a*	120	15 (12.5)	4 (3.3)	9 (7.5)	78 (65.0)	14 (11.7)
*GmF3′H* and *GmF3′5′H*-*b*	48	9 (18.8)	7 (14.6)	17 (35.4)	11 (22.9)	4 (8.3)
*gmf3′h*-*b* and *gmf3′5′h*	22	20 (90.9)	2 (9.1)	0	0	0

## Discussion

Previous studies showed that *GmF3′H* and *GmF3′5′H* had at least two alleles, one (designated *GmF3′H* and *GmF3′5′H*-*a* in this study) encoding the whole amino-acid sequences and the other (designated *gmf3′h*-*b* and *gmf3′5′h* in this study) encoding prematurely terminated sequences due to a frame-shift (Zabala and Vodkin 2003; Zabala and Vodkin 2007). These two kinds of alleles of *GmF3′H*/*GmF3′5′H* were associated with the *T/W1* loci, respectively (Toda et al. 2002; Zabala and Vodkin 2007). In our research, four alleles including two novel alleles of *GmF3′H* and three alleles including one novel allele of *GmF3′5′H* were identified in soybean accessions. Among these, one allele of *GmF3′H* (*gmf3′h*-*a1*) also encoded a premature termination due to a single base deletion of adenine at coding sequence position 973 relative to the start codon (Fig. 2). The site of the base deletion in *gmf3′h*-*a1* was in front of that in the allele of *GmF3′H* identified previously, resulting in an earlier occurrence of the frame-shift. The other novel alleles identified in this study (*gmf3′h*-*a2* and *GmF3′5′H*-*b*) resulted from non-synonymous nucleotide substitutions. Association analysis of allele and phenotype showed that these two alleles had no influence on the corresponding gene.

Although the genetic diversity of *G. soja* was reduced 50 % by the bottleneck of domestication (Hyten et al. 2006), it appears that selection for *GmF3′H* and *GmF3′5′H* did not cause erosion of diversity. This was inferred by the finding of four *GmF3′H* alleles among cultivated soybeans, whereas *G. soja* only contained the *GmF3′H* allele. With regard to *GmF3′5′H*, nearly all wild soybeans (92.2 %) contained the *GmF3′5′H*-*a* allele while three *GmF3′5′H* alleles occurred among cultivated soybeans. The distribution of these alleles in cultivated and wild soybean was similar to an artificial selection locus *GmTfl1*, which is associated with growth habit (Tian et al. 2010). These results suggested that the *GmF3′H* and *GmF3′5′H* loci might also have undergone artificial selection along with wild soybeans to landraces and then cultivars. However, this kind of selection might go with the selection of seed coat color since these two genes are all associated with seed coat color.

Functional marker development is very important for gene discovery in crops. A polymorphism study of the *IFS1* and *IFS2* genes indicated that three SNPs in *IFS1* and two SNPs in *IFS2* were closely associated with all individual types and total seed isoflavone concentrations (Cheng et al. 2008). In the present study, gene-tagged markers of *GmF3′H* and *GmF3′5′H* were developed and verified. Analysis of the genotype in 272 soybeans including the partial mini core collection showed that *GmF3′H* was correlated with pubescence color and *GmF3′5′H* was correlated with flower color. Therefore, these gene-tagged markers can be used to genotype the cultivars on the target locus. By PCR and/or digestion, the alleles of *GmF3′H* and *GmF3′5′H* could be clearly separated, indicating that nearly all SNPs or InDels could be used for developing CAPS/dCAPs markers. However, the phenotypes of some accessions were still not correlated with the genotypes, differing from previous reports (Toda et al. 2002; Zabala and Vodkin 2007). This was due to the existence of other loci controlling pubescence, flower and seed coat color in soybean and/or the existence of other alleles of these two genes. For example, another locus *Td* is also related to the control of pubescence color in soybean, and some of the cultivars may have the *Td/td* allele that is near grey as exemplified in this study. All wild soybeans possessed the *GmF3′H* allele, indicating that the *Td* locus may not have effect in the *G. soja* accessions. In addition, the dominant *I* allele exhibits a completely colorless seed coat phenotype due to dominance inhibition possibly via a posttranscriptional mode of gene silencing (Senda et al. 2004). The *T* and *R* loci determine specific seed coat color only in combination with the recessive *i* allele. Therefore, this interaction also reduced the frequency of *GmF3′H* alleles in the surveyed accessions. Using partial mini core collection, three new alleles, *gmf3′h*-*a1*, *gmf3′h*-*a2* and *GmF3′5′H*-*b,* were identified, and their frequencies were 4.4, 2.6, and 23.2 %, which indicated that the materials we used for genotyping had abundant genetic variation.

## Electronic supplementary material

Below is the link to the electronic supplementary material.

Supplementary material 1 (DOC 3645 kb)
