# Supplementary material for: Allele-specific marker development and selection efficiencies for both flavonoid 3′-hydroxylase and flavonoid 3′,5′-hydroxylase genes in soybean subgenus soja
Source: Theor Appl Genet. 2013 Mar 6;126(6):1445–55. doi: 10.1007/s00122-013-2063-3 (PMC3664743; doi:10.1007/s00122-013-2063-3)
Supplement: Supplementary file 1 — Supplementary material 1 (DOC 3645 kb) [file 122_2013_2063_MOESM1_ESM.doc]

**Supplemental Fig. 1 Phenotype of different kinds of pubescence, flower and seed coat color in soybean.**

Two kinds of pubescence color (a, gray and tawny), flower color (b, purple and white) and five kinds of seed coat color (c, yellow, green, brown, black and double) in soybean were classified according to different colors.

Supplemental Table 1 Primers using for sequencing in this study

| Primer name | Sequence (5’->3’) |
| --- | --- |
| F3’H-1 | CATAGCACAAAATCAACCATGTCT |
| F3’H-2 | GGAAGAAAAACTCCAAAATGTAGAA |
| F3’He1-1 | CACATAACTACATCATCTCAAAACCTA |
| F3’He1-2 | CCGCTTTATATCTCTTGAACTATACAT |
| F3’He2-1 | GCGTAAAAGCTGCATCTTGTAA |
| F3’He2-2 | CTACCAAAAATGATTATTACCAAAGA |
| F3’He3-1 | TCCAACTACAACATCTCACCTTAGAA |
| F3’He3-2 | ACTAATCATTGAATCCCATCCATA |
| F3’He1-1 | GTGCCTTGCGTGATGAACTAC |
| F3’5’H-1 | GCCTCTAGAATGGACTCATTGTTACTTCTAA |
| F3’5’H-2 | CATGAGCTCCAACCAATTCTAAGAAATGTAA |
| F3’5’He1-1 | GTGCCTTGCGTGATGAACTAC |
| F3’5’He1-2 | CTCCGAATCTAACCTCTTCATAAGTT |
| F3’5’He2-1 | CAAACCAAATGTTTCAGGGTCTT |
| F3’5’He2-2 | CCATGGTAGTTATCCTCATATAACAAAT |
| F3’5’He3-1 | CCTCGCCTCATAATCATACCAC |
| F3’5’He3-2 | GTAGCCACAGCCACAAAATCA |
